# (-)-Epicatechin and inflammation: Unraveling the role of microbial metabolites and interindividual metabolic variability

**DOI:** 10.3389/fmicb.2026.1803543

**Published:** 2026-03-25

**Authors:** Hao Yu, Meiyi Ning, Baozhou Long, Mengyi Ren, Cheng Wang, Yunshu Wang, Hong Chen, Daiwen Chen, Ning Jiang, Chen Liu

**Affiliations:** 1 College of Light Industry and Food, Nanjing Forestry University, Nanjing, China; 2 Tea Resources Utilization and Quality Testing Key Laboratory of Sichuan Province, College of Horticulture, Sichuan Agricultural University, Chengdu, Sichuan, China; 3 Institute of Agricultural Products Processing, Jiangsu Academy of Agricultural Sciences, Nanjing, China; 4 College of Food Science, Sichuan Agricultural University, Ya’an, China; 5 Institute of Animal Nutrition, Sichuan Agricultural University, Chengdu, Sichuan, China

**Keywords:** anti-inflammatory mechanisms, bioavailability, colonic metabolites, gut microbiota, interindividual variability

## Abstract

(-)-Epicatechin (EC), a dietary flavan-3-ol abundant in tea, cocoa, and certain fruits, has demonstrated promising anti-inflammatory activity. This review summarizes the chemical structure, absorption, distribution, metabolism, and excretion (ADME) characteristics, and molecular mechanisms through which EC modulates inflammation. EC acts both directly—via suppression of factor-kappa B (NF-κB), mitogen-activated protein kinase (MAPK), and Janus kinase/signal transducers and activators of transcription (JAK/STAT) pathways—and indirectly through activation of nuclear factor erythroid 2-related factor 2 (Nrf2)-driven antioxidant responses that mitigate inflammatory damage. Importantly, we highlight recent evidence on colonic microbial metabolites of EC, which exhibit diverse and sometimes enhanced bioactivities compared to the parent compound. These low-molecular-weight phenolics participate in immune regulation, redox balance, and intestinal barrier protection, and thereby contribute substantially to the overall anti-inflammatory effects of EC. A core innovation of this review is to highlight that interindividual variability in EC metabolism, largely governed by gut microbiota composition and host enzymatic capacity, generates distinct metabolite profiles. These metabotype-dependent differences provide a mechanistic basis for interindividual variability in the anti-inflammatory efficacy of EC and should be carefully considered when interpreting experimental and clinical findings on EC. Finally, we discuss strategies to enhance EC’s bioavailability, including delivery system optimization and structural modification. This review offers mechanistic and translational insights into EC’s anti-inflammatory potential and highlights the need to integrate microbial metabolism and host factors into future dietary interventions.

## 1 Introduction

Chronic inflammation is increasingly recognized as a critical contributor to the onset and progression of numerous non-communicable diseases, including cardiovascular disorders, diabetes, neurodegeneration, and various cancers (Nakadate et al., 2025). Although conventional anti-inflammatory medications are effective in acute contexts, their chronic use is frequently hindered by side effects and declining efficacy. Consequently, attention has turned toward dietary bioactive compounds—particularly (poly) phenols—as promising alternatives owing to their multitargeted biological activities and favorable safety profiles (Barik et al., 2025). (Poly) phenols can modulate oxidative stress, inflammatory signaling, immune responses, and epithelial barrier integrity, thus offering substantial potential in the prevention and management of chronic inflammatory diseases.

Among dietary (poly) phenols, EC, a cis-configured flavan-3-ol abundant in tea, cocoa, apples, and berries, has emerged as a notable candidate due to its distinctive chemical properties and biological activities. EC’s structure features multiple phenolic hydroxyl groups, particularly on its B-ring, granting it potent antioxidant capabilities through efficient hydrogen donation and electron-transfer processes. These structural attributes enable EC to directly scavenge reactive oxygen species (ROS) and stabilize free radicals via resonance delocalization. Beyond direct radical scavenging, EC influences cellular redox status by activating endogenous antioxidant defense mechanisms, notably the Nrf2–ARE signaling pathway (L Suraweera et al., 2020). Given that oxidative stress serves as a critical upstream driver of inflammatory processes, exacerbating pro-inflammatory signaling cascades and cytokine release, EC’s antioxidant functions effectively disrupt the amplification loop between oxidative damage and inflammation. Additionally, EC can modulate inflammation by regulating redox-sensitive signaling pathways such as NF-κB and MAPKs, thereby attenuating inflammatory gene expression, immune cell activation, and tissue damage (Hong et al., 2022). Collectively, these structural and functional characteristics provide a compelling mechanistic basis for EC’s potential as an anti-inflammatory dietary intervention.

Despite substantial interest in EC’s anti-inflammatory effects, the majority of mechanistic studies have primarily examined the parent compound without fully considering its extensive *in vivo* metabolic transformations. EC demonstrates relatively low and variable oral bioavailability due to rapid phase II conjugation in the small intestine and liver, coupled with extensive microbial degradation in the colon (Oketch-Rabah et al., 2020). These ADME characteristics imply that bioactive forms present in systemic circulation often differ significantly from the ingested compound, consisting instead of complex mixtures of conjugated and microbial-derived metabolites. Of particular importance are the colonic microbial metabolites—low-molecular-weight phenolic derivatives generated via gut microbiota—which have recently attracted attention for their potential role in modulating inflammation (Marques et al., 2024). However, the precise anti-inflammatory mechanisms and relative contributions of these microbial metabolites remain poorly defined.

Moreover, considerable interindividual variability in EC metabolism exists, predominantly driven by differences in gut microbiota composition and host enzymatic profiles (Liu et al., 2024). Such variability can result in distinct metabolite signatures across individuals, potentially causing substantial divergence in biological outcomes following EC intake. Yet, the implications of these personalized metabolic profiles for inflammatory responses and nutritional interventions have rarely been comprehensively addressed in existing literature.

To bridge these critical knowledge gaps, this review systematically explores the anti-inflammatory mechanisms of EC, emphasizing the interplay between its structural properties, metabolic fate, and the bioactivities of its microbial metabolites. Furthermore, we highlight the importance of interindividual differences in EC metabolism and discuss their relevance for developing personalized nutritional strategies. Through this integrative approach, we aim to provide novel insights into the biological and clinical relevance of EC and its metabolites, thereby informing future translational research and dietary interventions targeting chronic inflammation.

## 2 Chemical structure and ADME characteristics of (-)-epicatechin

### 2.1 Structural characteristics and bioavailability

#### 2.1.1 Chemical structure of EC and its relevance to bioactivity

Catechins are a group of naturally occurring flavan-3-ols widely distributed in plant-derived foods and beverages. Structurally, they share a characteristic C6–C3–C6 framework comprising two aromatic rings (A and B) and a heterocyclic C ring, which typically adopts a pyran configuration (Parveen et al., 2025). Variations in the stereochemistry at the C2 and C3 positions of the C ring, as well as the oxidation state and hydroxylation pattern of the B ring, give rise to different catechin isomers. Among them, trans-catechins and cis-epicatechins represent two major geometric forms, each of which can occur in (+) or (-) enantiomeric configurations. In addition, (-)-catechin can undergo esterification with gallic acid (GA) to generate a series of gallate derivatives, including (-)-catechin-3-O-gallate, epicatechin-3-O-gallate (ECG), (-)-epigallocatechin-3-O-gallate (EGCG), and (-)-gallocatechin-3-O-gallate (Chen et al., 2022; Sato et al., 2022). Among the various catechin isomers, EC is one of the most abundant and biologically relevant forms. It is widely distributed in commonly consumed plant-derived foods such as tea, cocoa, apples, and berries, often accounting for a major proportion of the total flavan-3-ols present in these sources (Liu C. et al., 2022). Due to its high natural abundance and well-documented physiological activities, EC has become one of the most extensively studied catechins in nutritional and biomedical research. It is a white crystalline solid, highly soluble in water and methanol (Gadkari and Balaraman, 2015). Its C ring adopts a stable chair conformation within a heterocyclic benzopyran structure. The specific stereochemistry at the C2 and C3 positions defines it as a cis-type catechin, while the lack of a galloyl group at the C3 position classifies it as a non-galloylated form (Rocha et al., 2025; Chen S. et al., 2024).

The unique structural features of EC confer potent antioxidant properties, primarily through its multiple phenolic hydroxyl groups, which readily donate hydrogen atoms to neutralize free radicals, forming resonance-stabilized phenoxyl radicals (Daussin et al., 2021; Kostiæ et al., 2023; Imran et al., 2023). These hydroxyl groups not only play a central role in direct radical scavenging, but also modulate intracellular signaling pathways involved in oxidative stress and inflammation (Si et al., 2021). Notably, both the number and position of hydroxyl groups on the B ring are critical to its redox activity, with increased hydroxylation enhancing electron-donating capacity and thereby improving antioxidant efficacy (Chen et al., 2023; Platzer et al., 2022). Building upon these structure–function relationships, the following sections provide a systematic overview of the ADME characteristics of EC, along with its anti-inflammatory bioactivities, to further elucidate its mechanisms of action and potential health applications.

#### 2.1.2 Bioavailability, absorption, metabolism, and tissue distribution of EC

It is well recognized that phenolic compounds generally exhibit low bioavailability, with only a limited fraction of the ingested dose reaching systemic circulation (Di Lorenzo et al., 2021). As a result, a substantial proportion of tea (poly) phenols—including EC—pass through the small intestine largely unabsorbed and reach the colon intact (Shi et al., 2022). Compared to galloylated catechins such as EGCG, EC demonstrates relatively better bioavailability due to its simpler structure. However, its overall oral bioavailability remains low, with estimates of systemic absorption reaching only approximately 30% (Barrera-Reyes et al., 2021; Di Pede et al., 2023).

##### 2.1.2.1 Absorption

The absorption kinetics of EC have been widely investigated in both humans and animal models. Following oral administration of green tea extract (630 mg), plasma EC is rapidly absorbed, with T_max occurring at approximately 2 h (Day 1: 2.00 h; Day 15: 2.03 ± 0.13 h) (Park et al., 2018). The peak plasma concentration (C_max) (Daussin et al., 2021) typically falls between 0.3 and 1.0 μmol/L (Liu et al., 2021), while the area under the curve (AUC) varies considerably depending on the dose and formulation. Despite its relatively better bioavailability compared to galloylated catechins such as EGCG, EC still exhibits moderate systemic exposure, with an average oral bioavailability of approximately 30% (Di Pede et al., 2023). These pharmacokinetic parameters are influenced by multiple factors, including species differences, individual variability, dosage, and the presence of dietary matrices. In beagle dogs, oral administration of 173 mg green tea catechins led to plasma levels of EC-glucuronide and EC-sulfate with median concentrations of 0.2 and 1.0 μmol/L, respectively (Mata-Bilbao et al., 2008). In rats, a high oral dose of tea (poly) phenols (700 mg/kg) yielded an EC C_max of approximately 0.96 μg/mL and a T_max of 40 min (Zhang et al., 2015). These findings collectively suggest that EC is absorbed rapidly but inconsistently, with considerable interspecies and interindividual variation in systemic exposure.

##### 2.1.2.2 Tissue distribution

After oral administration, EC shows limited systemic distribution and is largely retained in the gastrointestinal tract. Studies have reported that approximately 98% of the administered EC remains within the gut shortly after ingestion (Borges et al., 2016), while only a small fraction reaches peripheral tissues such as the liver, kidneys, lungs, heart, brain, and muscle.

In a radiolabeled study using (2-^14^C)-EC in rats, less than 0.1% of the administered dose was detected in the brain, testes, spleen, heart, skeletal muscle, and lungs. Slightly higher levels of radioactivity (0.2–1.0%) accumulated in the liver and kidneys, but most extraintestinal tissues showed a rapid decline in EC levels to below 0.1% within 24 h post-administration, and further reductions were observed by 48 h (Borges et al., 2016).

##### 2.1.2.3 Metabolism

The metabolism of EC plays a pivotal role in determining its overall bioavailability and biological activity. In particular, microbial metabolism in the gut is increasingly recognized as a key contributor to EC’s systemic effects, as it generates low-molecular-weight metabolites (LMWMs) with distinct bioactive profiles (Liu et al., 2024). Specifically, EC undergoes two major types of metabolic transformations: (1) phase II conjugation reactions mediated by host enzymes, and (2) microbial degradation occurring primarily in the colon. Both pathways contribute to shaping the pharmacokinetic behavior and functional outcomes of EC *in vivo*.

###### 2.1.2.3.1 Phase II conjugation reactions

Within one hour of ingestion, approximately 98% of the administered EC remains in the gastrointestinal tract, gradually shifting toward the distal intestine over time, where extensive phase II metabolism occurs (Borges et al., 2016; Wang et al., 2018b). Phase II metabolism, also known as conjugation, typically involves the covalent attachment of endogenous molecules—such as glucuronic acid, sulfate, or methyl groups—to nucleophilic functional groups on the substrate, including hydroxyl, carboxyl, or amino groups. These reactions are catalyzed by a variety of enzymes, notably UDP-glucuronosyltransferases (UGTs), sulfotransferases (SULTs), catechol-O-methyltransferase (COMT), glutathione S-transferases, and N-acetyltransferases (Higgins and Hayes, 2011; Miners et al., 2010; Sim et al., 2012). Tissue-specific enzyme activity studies have revealed that the upper small intestine, cecum, and proximal colon exhibit the highest UGT activity, identifying the small intestine as the primary site for EC glucuronidation. In contrast, sulfated metabolites of EC are found almost exclusively in bile, indicating that the liver is likely the major, if not the sole, site for sulfation. The liver also exhibits the highest COMT activity, suggesting that it is the predominant organ responsible for methylation of EC’s catechol moieties. Notably, COMT enzymes are most active in the liver and kidneys, but are also present in the pylorus of the stomach and in the small intestine, where they participate in the methylation of available phenolic substrates (López-Yerena et al., 2021).

Glucuronidation is the predominant phase II metabolic pathway for tea (poly) phenols. This reaction is catalyzed by UGTs, which transfer glucuronic acid from the co-substrate uridine 5’-diphospho-α-D-glucuronic acid to nucleophilic functional groups on the substrate, typically hydroxyl groups. The resulting β-D-glucuronide conjugates are more water-soluble and readily excreted (Guillemette et al., 2014). Studies have shown that EC undergoes glucuronidation in rat liver microsomes, forming detectable glucuronide metabolites. However, similar metabolic conversions were not observed in human liver or intestinal microsomal preparations (Xiao and Hogger, 2013). This species-specific difference highlights potential limitations in extrapolating EC metabolic data across models and underscores the need for careful interpretation when comparing results from animal and human studies.

Another important phase II metabolic pathway of EC is sulfation, which is particularly active in the liver. This process is catalyzed by SULTs, which transfer a sulfate group from the donor molecule 3’-phosphoadenosine-5’-phosphosulfate to nucleophilic acceptor groups, typically hydroxyl or amine functionalities. Sulfation represents a major route for EC metabolism in both the human liver and intestine. Among the 13 known human cytosolic SULT isoforms, SULT1A1, SULT1A3, and SULT1C4 exhibit sulfating activity toward catechins, whereas other isoforms contribute minimally (Mei et al., 2021). Radiotracer-based metabolomic studies have further revealed striking interspecies differences in EC sulfation. In humans, sulfated metabolites constitute the predominant circulating forms. In contrast, rats exhibit no detectable sulfate conjugates, with glucuronides representing the major metabolites, whereas mice display only minor sulfation. These findings demonstrate that sulfation efficiency is substantially higher in humans, underscoring species-specific enzymatic differences that are critical for accurate preclinical-to-human extrapolation (Ottaviani et al., 2016).

Methylation of EC is primarily catalyzed by COMT, which transfers a methyl group from S-adenosylmethionine to the catechol moiety of flavan-3-ols. In simple catechins, this reaction typically occurs at the 3’ and 4’ positions on the B ring, yielding mono- or di-methylated metabolites (Docampo-Palacios et al., 2020). *In vitro* studies have demonstrated that non-gallated catechins, such as EC, are highly efficient substrates for COMT, whereas gallated catechins are methylated far less efficiently. Consistent with these observations, *in vivo* metabolic profiling in rats has revealed that EC undergoes extensive mono- and di-methylation, often followed by subsequent glucuronidation or sulfation, resulting in the formation of structurally diverse phase II conjugates (Shang et al., 2017). Structural analysis revealed methylation at the 3’, 4’, and 4” hydroxyl positions, indicating that COMT preferentially targets not only the B-ring catechol group but also additional hydroxyls on the galloyl moiety. The identification of 3’,4”-di-O-methyl-(-)-epicatechin gallate further suggests a sequential methylation process, highlighting the enzymatic flexibility of COMT in modifying multiple hydroxyl sites within a single molecule.

**FIGURE 1:**
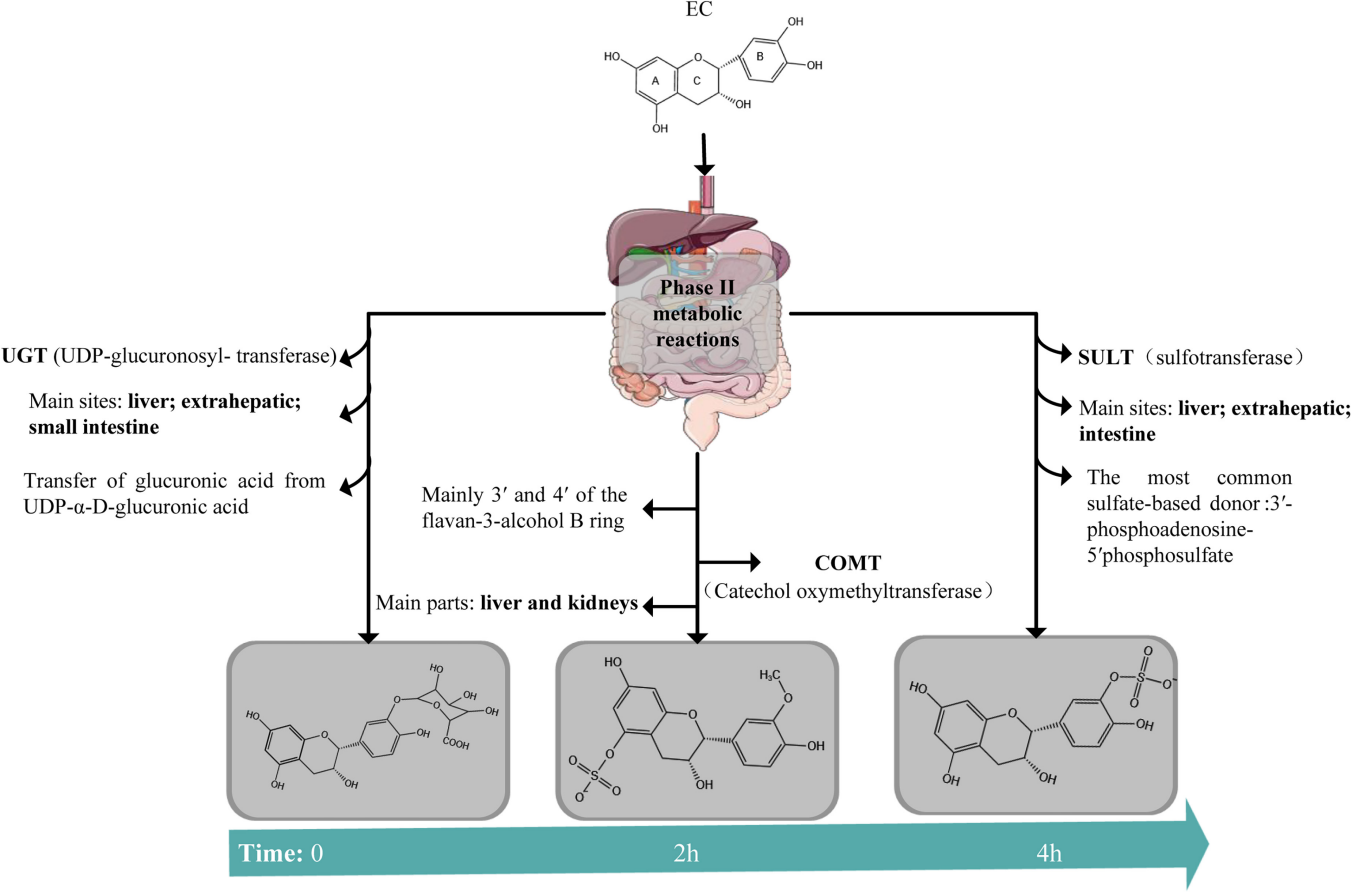
Schematic representation of the chemical structure of EC and its major phase II metabolic conjugation pathways, including glucuronidation (via UGT), methylation (via COMT), and sulfation (via SULT).

Conjugation reactions—including glucuronidation, sulfation, and methylation—significantly alter the physicochemical properties of flavan-3-ols, thereby influencing their plasma retention, excretion kinetics, and biological activity. Among them, glucuronidation and sulfation typically lead to a marked reduction in bioactivity due to the masking of free hydroxyl groups. In contrast, the effects of methylation appear more complex: while it reduces the number of available hydroxyl groups, it simultaneously increases lipophilicity, potentially enhancing passive diffusion across cell membranes (Monagas et al., 2010). A human study reported that 20 ± 2% of ingested EC was recovered as structurally related EC metabolites (SREMs) in urine within 48 h. Several metabolites, including 3’-O-methyl-(-)-epicatechin-5-O-glucuronide, 3’-O-methyl-(-)-epicatechin-7-O-glucuronide, (-)-epicatechin-7-sulfate, and (-)-epicatechin-7-O-glucuronide, were detectable in plasma but not in urine, suggesting either extended systemic retention or alternative elimination routes. This highlights the role of conjugation in modulating EC’s metabolic fate and possibly enhancing its systemic availability. Ottaviani et al. further demonstrated that although O-glucuronidation of EC could theoretically occur at five different hydroxyl positions, only a limited number of glucuronide isomers—primarily (-)-epicatechin-3’-β-D-glucuronide—were predominant in plasma. Along with (-)-epicatechin-3’-sulfate (E3’S) and 3’-O-methyl-(-)-epicatechin-5/7-sulfate, these three metabolites accounted for the major SREMs detected in human circulation (Ottaviani et al., 2012). Figure 1 provides a schematic overview of the chemical structure of EC and its principal conjugation pathways.

###### 2.1.2.3.2 Colonic metabolism of EC

Following ingestion, approximately 70% of EC reaches the colon, where it undergoes extensive microbial metabolism before absorption into systemic circulation (Ottaviani et al., 2012). A key initiating step is the reductive cleavage of the heterocyclic C-ring of EC, which dismantles the flavan-3-ol scaffold and enables downstream catabolism. This reaction is catalyzed by specific gut bacteria such as *Eggerthella lenta* rK3, which converts EC and related catechins into 1-(3,4-dihydroxyphenyl)-3-(2,4,6-trihydroxyphenyl) propan-2-ol through C-ring opening under anaerobic conditions (Kutschera et al., 2011). This intermediate is further metabolized into 5-(3’,4’-dihydroxyphenyl)-γ-valerolactone (3,4-DHPV), a hallmark microbial metabolite of flavan-3-ols and a widely recognized biomarker of EC and related polyphenol intake (Di Pede et al., 2023). Quantitative studies have shown that 3,4-DHPV can account for 50% or more of the originally ingested EC, highlighting its metabolic predominance and diagnostic potential for assessing EC exposure and microbial bioactivation efficiency (Mena et al., 2019a). 3,4-DHPV then undergoes lactone ring hydrolysis to yield 4-hydroxy-5-(3’,4’-dihydroxyphenyl) valeric acid and related isomers, such as 3’,4’-hydroxyphenylvaleric acid. These transformations are facilitated by a broader bacterial consortium, including *Flavonifractor plautii* aK2, which along with *E. lenta*, cooperatively modulates catechin catabolism in the distal gut.

**FIGURE 2:**
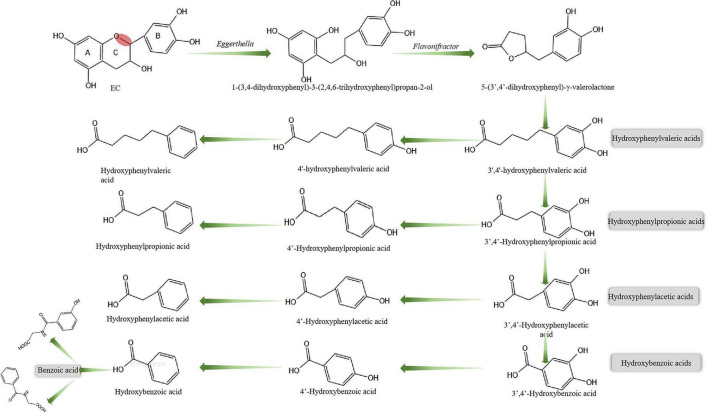
Schematic overview of the colonic microbial degradation pathways of EC.

Once valerolactones and phenylvaleric acids have been generated, further microbial degradation of EC proceeds through β-oxidation of the side chain, leading to the production of progressively smaller phenolic acids, such as hydroxyphenylpropionic acid, hydroxyphenylacetic acid, and hydroxybenzoic acid (Serra et al., 2011). Concurrently, dehydroxylation may occur at the 3’, 4’, or 5’ positions of the original B-ring, resulting in a wide range of structurally simplified phenolic metabolites. These transformations are mediated by a variety of colonic bacteria, including *Clostridium* spp., *Flavonifractor plautii*, and *Eubacterium ramulus* (Jin and Hattori, 2012). In an *in vitro* fecal fermentation model, Roowi et al. identified four major EC degradation products, accounting for approximately 32%–54% of the initial EC input. These included 3-(3-hydroxyphenyl)-propionic acid, 3,4-DHPV, 4-hydroxyphenylacetic acid and 5-(3,4-dihydroxyphenyl)-γ-valeric acid, confirming the ring-fission and chain-shortening activities of the gut microbiota (Roowi et al., 2010). *In vivo*, Stalmach et al. reported that approximately 70% of ingested EC could be recovered in the ileal fluids of human subjects—33% as intact catechins and 37% as microbial metabolites (Roowi et al., 2010). This indicates that a substantial portion of EC and its conjugates reaches the colon unabsorbed, where microbial enzymes cleave glycosidic linkages and initiate catabolic conversion into ring-opened and chain-shortened phenolic acids. Some of these compounds may undergo further transformations, including aromatic ring opening, leading to the generation of short-chain fatty acids (SCFAs) such as acetate and butyrate (Chen et al., 2020). A schematic representation of this microbial metabolic sequence is provided in Figure 2.

##### 2.1.2.4 Excretion

**FIGURE 3:**
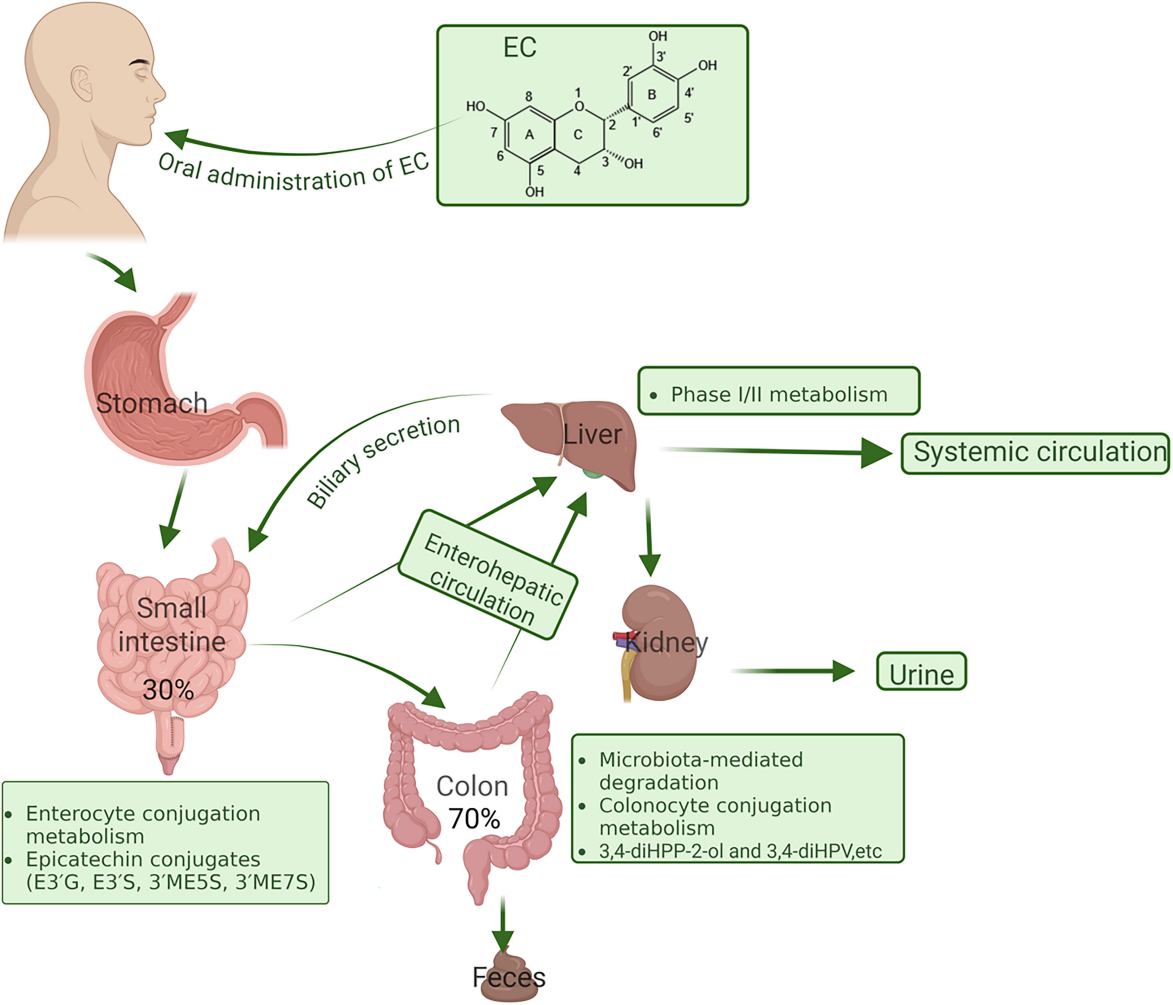
Schematic overview of the metabolic fate of EC following oral administration, illustrating its absorption, biotransformation, circulation, and excretion in the human body. Created in BIORENDER.com.

Urinary excretion represents the primary elimination route for EC and its metabolites in both humans and animals, with negligible amounts detected in feces. This pattern indicates that a substantial proportion of EC is either absorbed intact in the small intestine or converted into microbial metabolites in the colon, subsequently absorbed and systemically cleared via renal pathways (Mei and Chen, 2023). Following ingestion, EC undergoes extensive phase II metabolism—including glucuronidation and methylation—prior to excretion. In animal models, further studies have provided insight into the dose-dependent excretion dynamics of EC and its microbial metabolites. Recent human intervention studies have further confirmed that, following oral intake of EC, its SREM and the colonic microbiota-derived product 3,4-DHPV are extensively excreted in urine within 24 h, exhibiting a clear dose-dependent relationship with intake (Ottaviani et al., 2018; Ottaviani et al., 2019). Notably, the majority of valerolactone in urine existed in conjugated forms, suggesting that microbial-derived metabolites, once absorbed, undergo host phase II modification before renal clearance. Building on these findings, Ottaviani et al. confirmed the multi-step metabolism of EC in humans by isolating and identifying a wide array of methylated, sulfated, and glucuronidated metabolites (Ottaviani et al., 2016). These included (-)-epicatechin-3’-O-β-D-glucuronide, E3’S, as well as methylated derivatives such as 3’-O-methyl-(-)-epicatechin-5-sulfate and 3’-O-methyl-(-)-epicatechin-7-sulfate. Together, these metabolites provide compelling evidence for the extensive and complex biotransformation of EC within the human body. These findings collectively demonstrate that EC metabolism involves a coordinated sequence of intestinal absorption, hepatic and colonic biotransformation, and renal elimination, with distinct phase II conjugates acting as clearance intermediates and serving as characteristic metabolic signatures of EC exposure (Figure 3).

### 2.2 Factors influencing the bioavailability of EC

**FIGURE 4:**
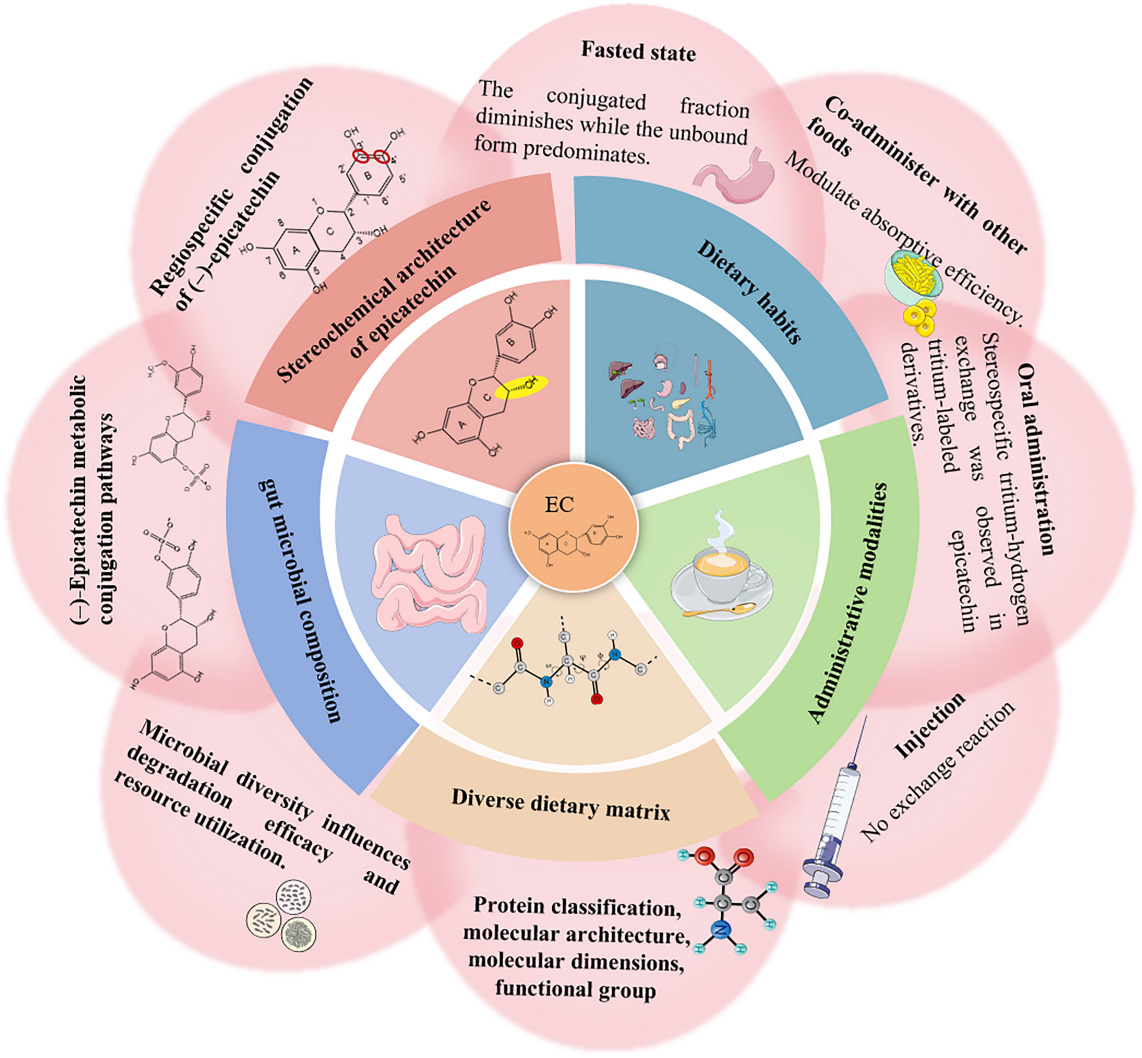
Overview of factors affecting the bioavailability and absorption of EC.

The bioavailability and absorption of EC are modulated by multiple factors, including its chemical structure (particularly stereochemistry and galloylation), the route and form of administration, dietary matrix components, gut microbial composition, and habitual dietary patterns (Figure 4). Structurally, the presence of a 3-O-galloyl moiety has been shown to significantly reduce intestinal absorption and increase biliary excretion, distinguishing non-galloylated catechins like EC from their galloylated counterparts (Liu et al., 2025). In a mechanistic study, Ávila-Avilés et al. highlighted that the nature and positional substitution of conjugated groups on the EC molecule critically influence its transport across the intestinal barrier (Ávila-Avilés et al., 2025). Specifically, these structural features determine whether EC is retained in enterocytes, effluxed back into the intestinal lumen, or successfully absorbed into systemic circulation.

The mode of administration also has a profound effect on the pharmacokinetics and systemic availability of EC. In a study by Catterall et al., tritiated EC was administered to rats either orally or intravenously at doses mimicking typical human dietary intake. Notably, oral administration led to a measurable exchange of tritium with hydrogen in body water within 3 h, a phenomenon absent after intravenous dosing (Catterall et al., 2003). This differential pattern suggests that EC undergoes metabolic cleavage in the gastrointestinal tract—likely mediated by gut microbiota—releasing tritium-labeled fragments capable of exchanging with body water. These findings point to a significant role for microbial activity in the initial transformation of EC following oral ingestion, underscoring the importance of the gut environment in determining its metabolic fate.

In addition, dietary components can significantly affect the absorption and overall bioavailability of EC by altering gastrointestinal conditions or interacting directly with the compound. Among these, proteins have drawn particular attention for their ability to bind (poly) phenols, including catechins, through hydrogen bonding and hydrophobic interactions. However, the strength and consequences of such interactions vary depending on protein type, molecular structure, size, and environmental factors such as pH and ionic strength (Yildirim-Elikoglu and Erdem, 2018). These interactions can either inhibit catechin absorption by forming insoluble complexes or protect catechins from degradation, depending on the specific food matrix. Overall, current evidence suggests that protein–catechin binding has the potential to modulate EC bioavailability, though the precise outcome remains context-dependent.

In addition to macronutrients like proteins, carbohydrates, and lipids, interindividual differences in gut microbiota composition also play a key role in shaping EC metabolism. These microbial variations influence enzymatic activities involved in ring fission and phenolic acid production, thereby contributing to person-specific bioavailability outcomes. A more detailed discussion of microbiota-related variability is presented in the following section.

### 2.3 Interindividual variability in EC metabolism and its impact on the anti-inflammatory effects of EC

Substantial interindividual variability exists in the metabolic fate and systemic availability of EC, largely driven by differences in gut microbial composition and host-related factors. Even under controlled intake conditions, marked disparities in plasma concentrations, urinary metabolite profiles, and the abundance of colonic metabolites have been repeatedly observed.

The gut microbiota, a complex and dynamic microbial ecosystem primarily residing in the colon and distal small intestine, is considered a key determinant of EC biotransformation. This microbial community comprises over 1 × 10^14^ cells and functions as an essential metabolic “organ” that interacts closely with the host (Braicu et al., 2013; Kasprzak-Drozd et al., 2021). Its composition is shaped by a multitude of factors, including delivery mode at birth, age, diet, antibiotic use, host immunity, stress, and environmental exposures (Peters et al., 2022; Chi et al., 2019; Manach et al., 2017). For example, vaginally delivered infants harbor microbiota rich in *Bifidobacterium*, *Escherichia coli*, and *Bacteroides*, whereas those born via cesarean section tend to be colonized by *Enterococcus*, *Streptococcus*, and *Staphylococcus* spp. (Shao et al., 2019; Roswall et al., 2021; Li et al., 2020). Similarly, breastfeeding favors the development of *Lactobacillus* and *Clostridium*-dominant communities, which differ significantly from those in formula-fed infants (Li et al., 2020).

These interindividual differences in microbiota composition lead to distinct EC metabolic phenotypes. Gabriele et al. demonstrated that when fecal samples from ten healthy individuals were subjected to *in vitro* colonic fermentation with mixed (poly) phenols, the resulting metabolite profiles were highly personalized, with no consistent correlation in AUC values across subjects. Some phenolic acids, such as 3-hydroxybenzoic acid, were detected exclusively in certain individuals, highlighting subject-specific metabolic capabilities (Gross et al., 2010). Likewise, Liu et al. reported striking variability in EC metabolism after *in vitro* co-incubation with fecal microbiota from 24 volunteers, with the number of detectable EC-derived metabolites ranging from 1 to 11, and a 76.7-fold difference in residual EC levels observed after 2 h of fermentation (Liu et al., 2020).

*In vivo* evidence further confirms these findings. Pedro et al. administered green tea and green coffee extracts to 11 individuals over 8 weeks and monitored urinary excretion of colonic EC-derived metabolites. Their results revealed three distinct “metabotypes” each characterized by the differential excretion of trihydroxyphenyl-γ-valerolactone, dihydroxyphenyl-γ-valerolactone, and hydroxyphenylpropionic acid (Mena et al., 2019b). Similarly, Lucas et al. noted variable EC absorption in the jejunum, while other studies reported wide interindividual differences in urinary levels of colonic metabolites (Actis-Goretta et al., 2013).

Apart from microbial influences, host-related genetic factors also contribute to variability in EC metabolism. Genetic polymorphisms in phase II metabolic enzymes—such as UGTs, SULTs, and COMT—may influence the efficiency of EC conjugation and clearance (Mei et al., 2021; Kurogi et al., 2021). These enzymes catalyze glucuronidation, sulfation, and methylation of EC, determining the balance between free and conjugated forms in circulation. Polymorphic variants in these genes can result in altered enzyme activity or expression, ultimately affecting EC’s systemic availability and biological activity. For instance, variations in COMT activity have been associated with differences in methylated catechin metabolites, which may display altered transport, tissue retention, and functional properties (Miller et al., 2012).

Such metabolic variability has functional implications. Certain EC-derived microbial metabolites, such as 3,4-DHPV and 3,4-dihydroxyphenylpropane-2-ol (3,4-DHPP-2-ol), possess enhanced biological activity compared to their parent compound. Augusti et al. demonstrated that 3,4-DHPV exerts antiproliferative effects in HT29 colorectal cancer cells (Augusti et al., 2021), while 3,4-DHPP-2-ol showed 1.8-fold greater antioxidant capacity than EC in the DPPH radical scavenging assay (EC_50_ = 5.97 μM) (Chen et al., 2020). Notably, Dufour et al. reported that 3,4-DHPV—but not EC—was capable of activating Nrf2 signaling in HepG2 cells, with an EC_50_ of 74.55 μg/mL (Dufour et al., 2022). Consistent with this finding, Liu et al. observed that treatment with 3,4-DHPV upregulated 14 and 10 Nrf2-associated proteins to a greater extent than EC in Hepa1c1c7 and Caco-2 cells, respectively (Liu C. et al., 2022). In contrast, EC induced only three and four such proteins in the two cell lines. These results collectively suggest that gut microbial metabolites of EC, particularly 3,4-DHPV and 3,4-DHPP-2-ol, may serve as the principal effectors mediating its antioxidant and cytoprotective activities. Therefore, elucidating the interindividual differences in EC metabolism—especially those shaped by gut microbiota—is critical for accurately interpreting dietary intervention outcomes and for advancing the development of personalized nutritional strategies aimed at maximizing the health benefits of EC.

Individual variations in the anti-inflammatory effects of EC are closely linked to differences in EC metabolism among individuals. It is precisely due to distinctions in EC metabolism during Phase II (glucuronidation, sulfation, methylation) and its conversion capacity by the gut microbiota that variations arise in its *in vivo* exposure levels and the composition of EC and its active metabolites. These variations further influence the regulatory intensity of inflammatory signaling pathways such as NF-κB and Nrf2, ultimately manifesting as differences in individual anti-inflammatory efficacy.

Among them, microbial metabolism plays a pivotal role in mediating the anti-inflammatory effects of EC. Given that the formation of colonic metabolites determines the biological activity of EC, inter- and intra-individual variability in gut microbiota composition can critically influence the types and amounts of metabolites produced (Hu et al., 2024; Li and Van de Wiele, 2023), thereby impacting the observed health outcomes (Liu et al., 2020; Liu M. et al., 2022; Favari et al., 2024). Lessard-Lord et al. investigated individual differences in EC microbial metabolism among 34 participants and classified them into “fast converters” and “slow converters” based on their EC conversion rates (Lessard-Lord et al., 2023). Fast converters exhibited higher production of short-chain fatty acids (SCFAs) and greater microbial metabolic efficiency. The study suggested that habitual consumption of flavanol-rich foods may enrich microbial taxa capable of efficiently metabolizing EC.

Overall, variability in gut microbiota composition can lead to significant differences in the generation of bioactive EC metabolites, ultimately affecting its anti-inflammatory efficacy. These findings underscore the importance of considering personalized nutrition strategies to optimize EC-related health benefits.

## 3 Molecular mechanisms of EC in anti-inflammatory pathways

Inflammation is a complex biological response mediated by intricate signaling pathways that coordinate cellular responses to injury, infection, or stress (Tavares et al., 2020). Various factors, including pathogenic infections, autoimmune disorders, environmental toxins, and physical injuries, can trigger inflammatory responses by activating immune cells and cytokine networks (Furman et al., 2019). Inflammation is often characterized by redness, swelling, heat, pain, and loss of function, which can lead to tissue damage and chronic disease if dysregulated (Devi et al., 2015). Among the key pathways implicated in inflammatory responses are the NF-κB (Khan et al., 2020), MAPK, JAK/STAT (Salas et al., 2020), and Nrf2 pathways, along with macrophage polarization (Li et al., 2025d), particularly the balance between M1 (pro-inflammatory) and M2 (anti-inflammatory) phenotypes (Cutolo et al., 2022). EC exerts significant anti-inflammatory effects by modulating these signaling cascades, which regulate immune responses, oxidative stress, cytokine production, and macrophage polarization, ultimately contributing to the suppression of inflammation. Representative studies on the anti-inflammatory activities of EC itself are summarized in Table 1.

**TABLE 1:** Summary of studies investigating the anti-inflammatory effects and underlying mechanisms of EC, including experimental models, target pathways, and key outcomes.

Model	Source and dose	Effect	Mechanism	References
*In vitro* model				
RAW 264.7 cell				
LPS-induced inflammation in RAW 264.7 cells	Extracted from *Enteromorpha clathrata* 100, 200 mg/kg	Inhibited the ability of macrophages to phagocytose green fluorescent microspheres; Reduced NO overproduction	Inhibiting the phosphorylation of the signal proteins (p65 and p38) involved in the MAPKs/NF-κB pathways	(Huang et al., 2022)
LPS-induced production of pro-inflammatory mediators in RAW264.7 cells	Extracted from litchi flowers 0.1, 0.2, 0.3 mg/mL	Diminished inflammatory response; Decreased production of pro-inflammatory mediators	Inhibiting the inactivation of NF-κB, MAPKs (ERK, JNK and p38) and JAK 2/STAT 3; Reducing IL-1β, IL-6 and TNF-α levels; Down-regulating the protein expression of iNOS and COX-2 as well as the production of NO and PGE2	(Yang et al., 2015)
RAW264.7 mouse macrophages	EC 150 μM	Elevated antioxidant activity; Decreased TNF-α, IL-1β, and IL-6; Increased SCFAs production SCFAs	Mediation of intestinal flora; Inhibition of NF-κB, MAPK signaling pathway; Intestinal barrier and regulation of immune homeostasis	(Mao et al., 2024)
Caco-2 cell				
Differentiated Caco-2 cells as a model of epithelial barrier	EC 1–10 μM	Improved intestinal permeability as well as intestinal barrier function	Preventing NADPH oxidase upregulation; Inhibit the formation of metabolic byproducts	(Wang et al., 2020)
A hyper-cholesterol diet to induce the loss of intestinal epithelial layer integrity in Caco-2 cells	EC 10 μM	Restored intestinal barrier integrity	Reducing derived oxide production to modulate pro-inflammatory signaling	(Deiana et al., 2017)
3T3-L1 adipocyte				
TNF-α exposed 3T3-L1 adipocytes	EC 0.5–10 μM	Reduced inflammatory response and insulin resistance	Preventing TNF-α-induced NF-κB, MAPKs and AP-1 activation; Inhibiting LPS-induced TNF-αrelease	(Vazquez-Prieto et al., 2012)
Palmitate exposed 3T3-L1 adipocytes	EC 0.1 μM, 1 μM	Decreased inflammation in visceral adipose tissue; Relief of endoplasmic reticulum stress	Reducing the macrophage infiltration, pro-inflammatory signaling, and cytokine/chemokine levels	(Bettaieb et al., 2016)
Other cells				
Monosodium Urate (MSU)-induced acute gouty arthritis model in THP-1 cell	EC 20, 40, 80 μM	Increased THP-1 cell viability; Reduced pro-inflammatory cytokines;	Inhibiting NLRP3 inflammasome and the NF-κB signaling pathway	(Wu et al., 2022)
High glucose and lipopolysaccharide-induced inflammation in renal proximal tubular cells	EC 1–10 μM	Decreased intracellular ROS, peroxide levels, and pro-inflammatory factors; Decreased levels of TNF-α, IL-6, and MCP-1	Preventing the increase in main pro-inflammatory mediators, adhesion molecules, as well as MAPKs and the diminution of ROS generation	(Cilleros et al., 2020)
LPS-induced inflammatory response in MAC-T cells	EC *In vitro*: 1.5, 7.5, 15, 30 mg/mL	Reduced inflammatory response and tissue damage; Inhibited the expression and activity of COX-2 and Inos; Reduced NO and prostaglandin synthesis.	Inhibiting the MAPK and NF-κB signaling pathways	(Ma et al., 2021)
***In vivo* model**				
Lipopolysaccharide (LPS)-induced				
LPS-induced renal inflammation in Male Sprague Dawley (SD) rats	EC 80 mg/kg	Inhibited inflammatory cell infiltration; Attenuated LPS-induced functional impairment; Improved renal function indices	Inhibiting of TLR4-NF-κB pathway and the NOX activation	(Prince et al., 2017)
LPS-induced inflammatory response in the mouse mammary gland	EC *In vivo*: 10, 20, 30 mg/kg	Reduced inflammatory response and tissue damage; Inhibited the expression and activity of COX-2 and Inos; Reduced NO and prostaglandin synthesis.	Inhibiting the MAPK and NF-κB signaling pathways	(Ma et al., 2021)
LPS-induced ALI in C57BL6/N mice	EC 15 mg/kg	Alleviated inflammatory lung injury	Inhibiting the expression of TNF-αand IL-6; Reducing the recruitment and accumulation of neutrophils; Suppressing the p38 MAPK–AP1 signaling	(Xing et al., 2019)
HFD-induced				
HFD-induced intestinal permeabilization in male C57BL/6J mice	EC 20 mg/kg	Improved intestinal permeability as well as intestinal barrier function	The activation of the redox-sensitive ERK1/2 signaling pathway	(Wang et al., 2020)
HFD-induced visceral adipose tissue inflammation in C57BL/6J mice	EC 20 mg/kg	Decreased inflammation in visceral adipose tissue; Relief of endoplasmic reticulum stress; Decreased small pleural effusions in pleurisy; Improved lung histopathology	Reducing the macrophage infiltration, pro-inflammatory signaling, and cytokine/chemokine levels	(Bettaieb et al., 2016)
HFD cause Type 2 diabetes mellitus (T2DM) in GK rats and Wistar rats	EC 40, 80 mg/kg/day	Reduced serum LPS levels; Reduced systemic oxidative stress, amelioration of hepatic tissue inflammatory injury	Inhibition of LPS-TLR4/NF-κB pathway; Remodeling of gut microbiota and regulation of metabolic disorders	(Zeng et al., 2024)
Other models				
MSU -induced gouty arthritis in male C57BL/6 mice	25, 50, 100 mg/kg	Decreased ankle edema; Improved ankle histopathology	Inhibiting NLRP3 inflammasome and the NF-κB signaling pathway	(Wu et al., 2022)
*Dermatophagoies farina* extract causes AD-like skin lesions in NC/Nga mice	Extracted from *Alpinia officinarum* 1, 5 μM	Reduced skin inflammation; Suppressed ear swelling; Reduced dermal and epidermal hyperplasia; Restored epidermal thickness	Inhibiting of MAPK phosphorylation (ERK and JNK), NF-κB, and STAT1	(Song et al., 2021)
Dextran sulfate sodium (DSS)-induced UC in C57BL/6J mice model	Extracted from grape seed 100, 200, 300 mg/kg	Decreased the disease activity index and colon macroscopic damage index scores; Reduced body weight loss; Relieved colon contracture and crypt damage	Reducing TNF-α, IL-6, NO, MPO and MDA; Suppressing the transcriptional activation of NF-κB	(Zhang et al., 2016)
Chronic constriction injury (CCI)-induced neuropathic pain models in male SD rats	Extracted from *Camellia japonica* 30 mg/kg	Mechanical abnormal pain reduction, cold nociceptive sensitization relief, and reduction in spontaneous pain behavior	Inhibiting the activation of MAPK, microglia and pro-inflammatory cytokines	(Lim et al., 2022)
Carbon tetrachloride-induced liver fibrosis in six-week-old male SD rats	Extracted from *Saccharum officinarum* L.	Reduced hepatic fibrosis; Improved liver function parameters; Reduced fibrosis markers and hepatic histopathologic repair	Preventing the phosphorylation of MAPK molecules	(Wang et al., 2018a)
Carrageenan-induced pleurisy in mice	Green tea extract (dosage unspecified)	Decreased pleural effusion and cellular infiltration; Repair of lung histopathology	Reducing STAT-1 activation; Decreasing ICAM-1 and iNOS expression	(Di Paola et al., 2005)
Cigarette smoke-induced lung inflammation in male rats	EC 15 mg/kg	Scavenged ROS; Inhibited lipid peroxidation; Restored cell viability	Activating the Nrf2 pathway; Suppressing oxidative stress	(Tian et al., 2021)
TBI in male C57BL/6 mice	EC 5, 15, 45 mg/kg	Reduced brain damage; Improved neurological function; Increased cell survival; Reduced neutrophil infiltration; Decreased ROS production	Activating the endogenous Nrf2-ARE signaling pathway	(Cheng et al., 2016)

### 3.1 NF-κB pathway

The NF-κB pathway serves as a pivotal regulator of inflammatory responses, activated by various stimuli such as pro-inflammatory cytokines (e.g., TNF-α and IL-1β), microbial components, and oxidative stress (Liu et al., 2017). Upon activation, NF-κB translocates to the nucleus and initiates the transcription of genes encoding pro-inflammatory mediators, including cytokines, chemokines, and adhesion molecules (Singh and Singh, 2020). Notably, EC has been reported to suppress NF-κB activation by inhibiting the degradation of IκBα, an essential inhibitory protein. This results in the downregulation of pro-inflammatory gene expression and subsequent attenuation of inflammatory responses (Olêdzka and Czerwińska, 2023).

For instance, in a rat model of Lipopolysaccharide (LPS)-induced renal dysfunction, EC treatment significantly reduced NF-κB p65 phosphorylation at Ser536 and lowered the nuclear-to-cytoplasmic p65 ratio, while preventing IκBα degradation, indicating its capacity to suppress NF-κB activation and mitigate renal inflammation (Prince et al., 2017). In another study, Huang et al. isolated six (poly) phenols from *Enteromorpha clathrata* and evaluated their effects in LPS-stimulated RAW264.7 macrophages. Among them, EC, along with (-)-epigallocatechin-3-O-gallate (EGCG) and epicatechin-3-O-gallate (ECG), significantly suppressed NO production and the expression of IL-1β and COX-2. Mechanistically, EC inhibited the phosphorylation of NF-κB p65, leading to reduced expression of its active form (p-p65), thereby attenuating the inflammatory response (Huang et al., 2022). Similarly, Wu et al. showed that EC reduced inflammation in THP-1 cells and in a murine model of gouty arthritis by downregulating the NF-κB/NLRP3 axis (Wu et al., 2022). EC inhibited p-p65 nuclear expression and suppressed NLRP3 inflammasome activation, resulting in decreased cytokine production and macrophage infiltration in inflamed synovial tissues.

RANTES, a chemokine upregulated during tissue injury, is transcriptionally controlled by the NF-κB pathway. Song et al. reported that epigallocatechin, a catechin related to EC, suppressed RANTES production by blocking p50 nuclear translocation in IFN-γ/TNF-α-stimulated HaCaT keratinocytes, further confirming NF-κB-dependent anti-inflammatory activity (Song et al., 2021). Ma et al. provided the evidence of EC’s anti-inflammatory effect *in vivo* in a mouse model of mastitis (Ma et al., 2021). EC downregulated the expression of pro-inflammatory cytokines (IL-6, TNF-α, IL-1β) and mediators (COX-2, iNOS) in LPS-stimulated MAC-T cells and mammary glands. Integrative transcriptomic and proteomic analyses revealed that EC exerts its effect via the TMEM35A–TMPO–NF-κB axis, where TMEM35A facilitates membrane translocation and interacts with TMPO to block downstream signaling.

Lastly, in a model of DSS-induced ulcerative colitis (UC), EC derived from grape seeds ameliorated intestinal inflammation by reducing TNF-, IL-6, NO, MPO, and MDA levels, while enhancing antioxidant enzyme activity (Zhang et al., 2016). Further reporter gene assays confirmed that EC’s therapeutic efficacy was mediated through inhibition of NF-κB activation and oxidative stress (Morrison et al., 2014).

### 3.2 MAPK

The MAPK pathway, comprising extracellular signal-regulated kinase (ERK), c-Jun N-terminal kinase (JNK), and p38 MAPK subfamilies (Braicu et al., 2019), plays a crucial role in the regulation of inflammatory cytokine production (Sun and Nan, 2016). ERK primarily regulates cell proliferation and survival (Lavoie et al., 2020), JNK is involved in stress responses and apoptosis (Solinas and Becattini, 2017), while p38 MAPK is essential for inflammatory cytokine production and immune responses (Canovas and Nebreda, 2021). EC modulates MAPK signaling by inhibiting p38 and JNK phosphorylation, leading to decreased production of IL-6, IL-1β, and TNF-α, thereby suppressing inflammation and tissue damage (Qu et al., 2021). In a study investigating EC attenuation of LPS-induced acute lung injury (ALI) in mice, the level of phosphorylated p38 was increased to a lesser extent in the lung tissues of the EC-treated group compared with that of the LPS group, and immunohistochemical analyses similarly corresponded to this result. It was concluded that the inflammatory inhibitory effect of EC on ALI was related to the blockage of the activation of the p38 MAPK-AP1 signaling pathway, presumably by directly binding to the active site of p38 and inhibiting its catalytic activity (Xing et al., 2019).

In neuropathic pain, activation of the MAPK signaling pathway in the dorsal root ganglion (DRG) promotes the synthesis of TNF-αand IL-1β, exacerbating neuroinflammation and enhancing pain signaling (Zhang Y. et al., 2019). To explore potential therapeutic interventions, Lim et al. isolated and enriched EC from C. *japonica* leaf extract and used it for the treatment of Chronic constriction injury (CCI)-induced neuropathic pain in rats. Their results showed that EC treatment significantly inhibited the phosphorylation of ERK, p38, and JNK in the DRG, thereby reducing MAPK pathway activation, suppressing transcription factor expression, and lowering the production of inflammatory mediators associated with injury perception, ultimately alleviating inflammation-related pain hypersensitivity in CCI rats (Lim et al., 2022). In another study, EC, as the major component of sugarcane (poly) phenols, contributed to protecting against CCl_4_-induced liver fibrosis in rats. In hepatic stellate cells, transforming growth factor-β1 (TGF-β1) markedly upregulated α-SMA expression, a key marker of fibrosis, through activation of the MAPK signaling pathway. However, treatment with sugarcane (poly) phenols reversed the TGF-β1-inducedα-SMA upregulation, accompanied by reduced phosphorylation of p38 and JNK1/2. These findings suggest that EC mitigates liver fibrosis by inhibiting TGF-β1-mediated MAPK pathway activation and subsequent inflammatory and fibrotic responses (Wang et al., 2018a). In studies using Caco-2 cells, EC was shown to inhibit deoxycholic acid-induced monolayer permeability by suppressing NADPH oxidase upregulation, reducing oxidant production, and attenuating activation of the redox-sensitive ERK1/2 signaling pathway (Wang et al., 2020). Consistently, Monica et al. demonstrated that EC effectively counteracted oxysterol-induced IL-8 overproduction, a key event mediated by the NADPH oxidase/p38 MAPK/NF-κB inflammatory signaling cascade, further supporting its anti-inflammatory potential at the intestinal epithelial level (Deiana et al., 2017).

### 3.3 JAK/STAT

The JAK/STAT signaling pathway plays a central role in cytokine-mediated immune responses and is often dysregulated in chronic inflammatory diseases (Lv et al., 2024). EC exerts anti-inflammatory effects by inhibiting JAK phosphorylation, thereby preventing STAT dimerization and nuclear translocation, ultimately downregulating pro-inflammatory cytokine expression and restoring immune homeostasis (Hamsalakshmi et al., 2022). In a study by Yang et al., EC pretreatment significantly inhibited LPS-induced phosphorylation of JAK2 and STAT3 in RAW264.7 macrophages, accompanied by reduced IL-6 production (Yang et al., 2015). These findings suggest that suppression of the JAK2/STAT3 axis is a key mechanism by which EC attenuates inflammatory responses in macrophages (Yang et al., 2015).

In addition to these models, *Morinda citrifolia* leaves, rich in EC and coumarin, have been shown to ameliorate leukemia by promoting apoptosis and inhibiting inflammation and angiogenesis. Mechanistic studies indicated that the extract suppressed the activation of JAK2, STAT3, and STAT5A, thereby delaying the progression of myeloid malignancies (Ahmadi et al., 2019).

### 3.4 Macrophage polarization

Macrophage polarization plays a crucial role in shaping inflammatory responses (Murray, 2017). Classically activated M1 macrophages exhibit a pro-inflammatory phenotype, characterized by high production of TNF-α, IL-6, IL-1β, and NO, contributing to pathogen clearance but also exacerbating inflammation (Dousdampanis et al., 2025; Tardito et al., 2019). In contrast, alternatively activated M2 macrophages produce anti-inflammatory cytokines such as IL-10 and TGF-β, promoting tissue repair and resolution of inflammation (Campitiello et al., 2024). Maintaining the balance between M1 and M2 phenotypes is essential for inflammatory responses (Alvarez et al., 2016). EC has been shown to influence macrophage polarization by shifting the balance toward the M2 phenotype, thereby exerting an additional anti-inflammatory effect (Sano et al., 2024). For instance, EC treatment alleviated TNF-α-induced inflammatory responses in 3T3-L1 preadipocytes, partly through the activation of PPARγ, a key transcription factor known to favor M2 macrophage polarization (Vazquez-Prieto et al., 2012). Although the study primarily focused on adipocyte inflammation, the findings indirectly suggest that EC may facilitate macrophage phenotype switching toward an anti-inflammatory profile, further supporting its role in attenuating chronic inflammation.

In a High fat diet (HFD)-induced model of adipose tissue inflammation and insulin resistance using C57BL/6J mice, EC treatment markedly reduced adipose tissue macrophage infiltration, as evidenced by decreased F4/80 and NOX2 protein levels (Bettaieb et al., 2016). It also suppressed activation of the NF-κB signaling pathway and lowered tissue levels of pro-inflammatory mediators, including TNF-α and MCP-1. Notably, EC inhibited HFD-induced NOX4 overexpression, thereby alleviating oxidative stress and reducing the expression and release of cytokines and chemokines. By mitigating NOX4-driven oxidative stress, EC effectively prevented the shift from the anti-inflammatory M2 phenotype to the pro-inflammatory M1 phenotype, ultimately disrupting the cycle of macrophage-driven adipose tissue inflammation (Bettaieb et al., 2016).

### 3.5 Nrf2

Additionally, the Nrf2 pathway is a critical regulator of cellular redox balance, primarily functioning as a master regulator of antioxidant defense mechanism (de la Vega et al., 2018). Upon activation, Nrf2 translocates to the nucleus, where it binds to the antioxidant response element (ARE) and induces the transcription of cytoprotective genes, including heme oxygenase-1 (HO-1), superoxide dismutase (SOD), and glutathione peroxidase (Tossetta and Marzioni, 2022). These enzymes scavenge reactive oxygen species (ROS), mitigate oxidative stress, and thereby indirectly suppress inflammatory responses.

EC has been shown to activate Nrf2 (Cheng et al., 2024), enhance the expression of antioxidant enzymes, and inhibit oxidative stress-induced inflammation (Qu et al., 2021). In a model of cigarette smoke extract-induced lung inflammation, Xue et al. showed that EC attenuated oxidative stress and NLRP3 inflammasome activation by activating Nrf2 (Tian et al., 2021). Inhibition or knockdown of Nrf2 abolished the antioxidant and anti-inflammatory effects of EC, highlighting the pivotal role of Nrf2 activation (Tian et al., 2021). Mechanistically, EC promoted Keap1 degradation and upregulated downstream antioxidant genes such as NQO1 and HO-1, thereby suppressing ROS accumulation and inflammation (Tian et al., 2021).

In the traumatic brain injury (TBI) model constructed by Cheng et al. (2016), both ROS and Keap 1 expression were decreased in the EC-treated group, while nuclear accumulation of Nrf2 and cytoplasmic expression of the phase II enzymes SOD1 and NQO1 were increased to protect against oxidative stress. This is due to the fact that oxidative stress conditions induced by infiltrating neutrophils cause Nrf2 to be released from the Keap1-Nrf2 complex and translocated to the nucleus, where it activates the Nrf2-ARE pathway. However, in Nrf2 knockout mice, EC did not show neuroprotective effects. Therefore, the Nrf2-ARE pathway may be the main pathway for the neuroprotective effects of EC. Oxidative stress which was involved in the study, is in turn closely related to inflammation, and the two interact in a variety of physiological and pathological processes. Oxidative stress directly damages cell membranes, proteins, and DNA, and this damage triggers a cascade response that activates inflammatory signaling pathways (e.g., NF-κB) and prompts immune cells to release pro-inflammatory factors (e.g., TNF-α, IL-6, IL-1β).

Consistent findings were reported in a TBI model, where EC treatment not only reduced ROS accumulation but also promoted Nrf2 nuclear translocation and upregulated cytoplasmic levels of antioxidant enzymes such as SOD1 and NQO1 (Cheng et al., 2016). These antioxidant effects were accompanied by a significant attenuation of neuroinflammation, reflected by decreased pro-inflammatory cytokine production and suppressed activation of inflammatory signaling pathways (Cheng et al., 2016). Notably, in Nrf2-knockout mice, EC failed to confer neuroprotective and anti-inflammatory benefits, further confirming that activation of the Nrf2-ARE pathway constitutes a key mechanism by which EC mitigates oxidative stress and inflammation. Given that oxidative stress triggers inflammatory signaling pathways such as NF-κB and MAPK, the ability of EC to activate Nrf2 and counteract oxidative stress provides an important indirect mechanism for suppressing inflammation.

### 3.6 Other anti-inflammatory mechanisms of EC

In addition to the major pathways discussed, EC may exert anti-inflammatory effects through, e.g., modulation of gut microbiota composition and suppression of adhesion molecule expression.

EC has been reported to promote the growth of beneficial microbiota and enhance the production of short-chain fatty acids (SCFAs), both of which contribute to systemic anti-inflammatory effects (Markandey et al., 2021; Sorrenti et al., 2020). Mao et al. (2024) found that microbial metabolites of EC reduced NO release, increased total SCFA levels, and favored the growth of acidophilic probiotics such as *Lactobacillus acidophilus*, which can secrete antimicrobial substances and suppress pro-inflammatory cytokines including TNF-α, IL-1β, IL-6, and PGE2 (Mao et al., 2024). Consistently, Zeng et al. observed that high-dose EC treatment significantly modulated gut microbiota in type 2 diabetic (T2D) GK rats, promoting the relative abundance of anti-inflammatory probiotic genera such as *Akkermansia* and *Lactobacillus* (Zeng et al., 2024), which are known to enhance gut barrier function and suppress inflammation.

Moreover, EC can inhibit the expression of adhesion molecules, limiting immune cell infiltration and subsequent inflammation (Zhang L. et al., 2019). David et al. demonstrated that EC pretreatment blocked high glucose- and LPS-induced upregulation of ICAM-1 and suppressed VCAM-1 expression in NRK-52E cells, contributing to its renoprotective and anti-inflammatory effects (Cilleros et al., 2020).

## 4 Role of microbial metabolism of EC in its anti-inflammatory effects

As described in previous sections, the bioavailability of EC is relatively low, with nearly half remaining unabsorbed in the small intestine and undergoing microbial metabolism in the colon to generate a series of low-molecular-weight metabolites (LMWMs) (Chen et al., 2020). This biotransformation significantly enhances the bioactivities of EC, as many LMWMs exhibit diverse and often superior biological functions, including anti-inflammatory properties (Ozdal et al., 2016). Table 2 summaries the microbial metabolites of EC that have demonstrated anti-inflammatory effects, along with their precursors, experimental models, and underlying mechanisms. Notably, metabolites such as valeric acid derivatives and phenyl-γ-valerolactones have been shown to modulate inflammatory signaling pathways (Cremonini et al., 2019). These compounds can attenuate immune responses by reducing the production of pro-inflammatory cytokines, inhibiting NF-κB activation (Mena et al., 2019a), and enhancing antioxidant defenses via activation of the Nrf2 pathway (Melrose, 2023). In addition, microbial-derived EC metabolites contribute to maintaining gut health by promoting beneficial microbial populations, which in turn help regulate systemic inflammation (Xiong et al., 2023).

**TABLE 2:** Summary of microbial metabolites of EC involved in anti-inflammatory activity.

Metabolite	Precursor	Model	Effect	Mechanism	References
1-(3’,4’-Dihydroxyphenyl)-3-(2’,4’,6’-trihydroxyphenyl)-propan-2-ol (DiHPP-2-ol)	EC	/	/	/	
DiHPP-2-ol	EC	/	/	/	
5-(3’,4’-dihydroxyphenyl)- γ-valerolactone (3,4-DHPV)	DiHPP-2-ol	Caco-2 cells, R1/3, HEK293	Suppressed TNF-α, IL-6, IL-1β; Inhibition of NF-κB protein expression; Enhanced antioxidant enzymes; Reduced the inflammatory response of intestinal epithelial cells	Activating Nrf2; Inhibiting NF-κB	(Baron et al., 2024)
3,4-DHPV	DiHPP-2-ol	Rat cortical microglia model	IL-1β, TNF-α, and NO release were decreased; IL-6, MCP-1, COX-2, and iNOS gene expression was suppressed	Activating Nrf2 by inhibition on Keap1 and promoting Nrf2 nuclear translocation	(Marcolin et al., 2025)
3,4-DHPV	DiHPP-2-ol	Rat intestinal epithelial IEC-6 cells	Reduced IκBαphosphorylation; Inhibits LPS-induced inflammation	Selective inhibition of Inhibiting IκBαphosphorylation in the NF-κB pathway	(Kim et al., 2020)
5-(3’,4’-Dihydroxyphenyl)- γ-valeric acid	3,4-DHPV	/	/	/	(Momma et al., 2023)
3,4-Dihydroxyphenylpropionic acid (3,4-DHPPA)	5-(3’,4’-Dihydroxyphenyl)-γ-valeric acid	HIRI mice (hepatic macrophages)	Suppressed pro-inflammatory cytokines; Reduced liver tissue damage	Inhibiting HDAC activity in macrophages; Inhibition of NF-κB pathway	(Li et al., 2022)
3,4-Dihydroxyphenylacetic acid (DHAA)	3,4-DHPPA	T2D mice	Decreased levels of pro-inflammatory factors LPS and IL-6; Increased levels of anti-inflammatory factor IL-10; Improved colonic histopathology and oxidative stress	Inhibiting phosphorylation of JNK and p38 MAPK; Reducing MLCK protein expression and MLC phosphorylation;Enhancing antioxidant enzyme activity	(Liu M. et al., 2022)
DHAA	3,4-DHPPA	HFD mice	Decreased levels of pro-inflammatory factors (e.g., LPS); Decreased inflammatory cell infiltration in adipose tissue	Activating the pentose phosphate pathway; Inhibiting the NF-κB pathway; Enhancing the activity of antioxidant enzymes (SOD, GSH)	(Chen W. et al., 2024)
3,4-Dihydroxybenzoic acid (DHPBA, Protocatechuic acid, PCA)	DHAA	MAFLD mice	Decreased body weight, liver fat vacuoles; Decreased pro-inflammatory factors IL-1β, TNF-α; Decreased MDA levels; Increased SOD activity	Suppressing *Enterococcus faecalis*; Inhibiting TLR2/NF-κB pathway and reducing IL-1β/TNF-α release	(Tan et al., 2023)
GA	PCA	J774A.1 Macrophage cell model, Gout arthritis model	Suppressed NLRP3 inflammasome; Decreased IL-1β, caspase-1 p20 release	Enhancement of Nrf2-mediated antioxidant defense; Inhibition of caspase-1 activation	(Lin et al., 2020)
Pyrogallol	GA	hUCMSCs in ALI model	Increased HO-1 expression; Boosted antioxidant defense	Enhancing anti-inflammatory potential via Nrf2	(Zhang et al., 2022)
Caffeic acid	Catechol derivatives	BV2 cells; PTZ epilepsy model; LPS epilepsy model	Inhibited TNF-α, IL-1β; Suppressed PERK-NF-κB pathway; Decreased inflammatory cell infiltration; Decreased ACOD1 enzyme activity in brain tissue	Modulating ACOD1-mediated PERK-NF-κB pathway; Covalently binds to ACOD1 and inhibiting its enzymatic activity	(Li et al., 2025b)

Among these metabolites, 1-(3’,4’-dihydroxyphenyl)-3-(2’,4’,6’-trihydroxyphenyl)-propan-2-ol (DiHPP-2-ol), predominantly detected in the proximal colon during EC degradation (Ma et al., 2025), was reported to possess stronger antioxidant capacity than EC itself (Chen et al., 2020). Gleńsk et al. focused on DiHPP-2-ol isolated from grape seed extract to investigate its antioxidant and antitussive potential. Three antioxidant assays, DPPH, ABTS, and FRAP, were used to evaluate DiHPP-2-ol and compare it with control compounds such as gallic acid (GA), pyrogallic gallic acid and EC. In the ABTS and DPPH assays, DiHPP-2-ol activity was slightly higher than that of catechin and EC, but lower than that of GA and pyrogallocatechin. Meanwhile, when DiHPP-2-ol was applied to the guinea pig ileum at a concentration of 10 μM, it significantly reduced the amplitude of histamine-induced ileal contractions by 55%, which the researchers hypothesized might be achieved by blocking the H1 histamine receptor. Such findings suggest a potential role for DiHPP-2-ol in the anti-inflammatory effects of EC (Gleńsk et al., 2019).

A key further microbial metabolite is 5-(3’,4’-dihydroxyphenyl)-γ-valerolactone (3,4-DHPV), which has been identified as one of the most representative and bioactive metabolites of EC. Baron et al. demonstrated that 3,4-DHPV exhibited anti-inflammatory activity across multiple cell models, including HEK293 (Nrf2 activation), R3/1 (NF-κB inhibition), and Caco-2 (pro-inflammatory cytokine suppression) cells (Baron et al., 2024). Mechanistically, 3,4-DHPV promotes Keap1 degradation, leading to Nrf2 activation and upregulation of downstream antioxidant enzymes (e.g., HO-1, NQO1, SOD1), thereby inhibiting TNF-α-induced NF-κB activation and reducing the production of pro-inflammatory mediators such as TNF-α, IL-6, and IL-1β, while maintaining redox homeostasis (Baron et al., 2024). Additionally, 3,4-DHPV was shown to reverse integrin-mediated adhesion signaling abnormalities and improve mitochondrial function under inflammatory conditions, highlighting its dual antioxidant and anti-inflammatory roles (Baron et al., 2024). Further evidence was provided by Marcolin et al. using primary rat cortical microglia, where 3,4-DHPV alone (25–50 μM) significantly suppressed Lipopolysaccharide (LPS)-induced release of IL-1β, TNF-α, and NO (Marcolin et al., 2025). Importantly, when combined with curcumin, 3,4-DHPV exhibited a strong synergistic anti-inflammatory effect at low micromolar concentrations (5 μM 3,4-DHPV + 5 μM curcumin), markedly reducing the expression of multiple pro-inflammatory genes (e.g., IL-6, MCP-1, COX-2, IL-1β, TNF-α, iNOS) and their secreted protein levels (Marcolin et al., 2025). Mechanistic studies indicated that the combination treatment synergistically inhibited NLRP3 inflammasome activation, suppressed NOX2 expression, and upregulated the Nrf2 signaling pathway (HO-1 and NQO1), thereby mitigating oxidative stress and neuroinflammation (Marcolin et al., 2025). Additionally, Kim et al. further synthesized and evaluated the enantiomers of 3,4-DHPV, (R)- and (S)-3,4-DHPV in IEC-6 intestinal epithelial cells (Hur et al., 2020). Their study revealed that (S)-3,4-DHPV exhibited a stronger inhibitory effect on LPS-induced inflammation by dose-dependently suppressing the phosphorylation of IκBα, reinforcing the anti-inflammatory potential of 3,4-DHPV through NF-κB pathway inhibition (Hur et al., 2020).

During the passage of 3,4-DHPV through the intestine, esterases (e.g., carboxylesterases) in the intestinal epithelium or serum can catalyze the ring-opening of the lactone ring of 3,4-DHPV, resulting in 5-(3’,4’-dihydroxyphenyl)-γ-valeric acid (γVA). Meanwhile, intestinal flora can express esterases or lactonases that directly hydrolyze 3,4-DHPV to γVA. Traditionally, the formation of EC metabolites such as γVA has been thought to depend on the conversion by gut microbiota (Ozdal et al., 2016; Lee et al., 2017; Mena et al., 2017). In a study by Momma et al., the view that γVA is produced only by gut microbes was broken by co-incubating human serum, HUVEC cell lysate, or PON-transfected HEK293FT cell lysate with 3,4-DHPV (1–500 μM) at 37°C, confirming that PON1/3 in serum can enzymatically hydrolyze 3,4-DHPV to γVA by enzymatic hydrolysis, forming a new pathway of host metabolism (Momma et al., 2023). However, there have been few studies on the anti-inflammatory effects of γVA alone, probably due to the fact that it exists more in plasma as phase II metabolites (e.g., sulfated, glucuronidated derivatives), which are difficult to detect and quantify. As EC undergoes progressive microbial degradation, a series of low-molecular-weight phenolic metabolites are produced, each contributing to anti-inflammatory activities through distinct mechanisms. Li et al. reported that 3,4-dihydroxyphenylpropionic acid (3,4-DHPPA), a major microbial metabolite of EC, suppressed pro-inflammatory cytokine production in hepatic macrophages during ischemia/reperfusion injury (HIRI). Mechanistically, 3,4-DHPPA inhibited histone deacetylase (HDAC) activity, highlighting macrophages as a key target of its anti-inflammatory effects (Li et al., 2022). Similarly, a few studies have found that hydroxyphenylacetic acid reduces systemic inflammation. Wang et al. demonstrated that treatment with hydroxyphenylacetic acid increased serum IL-10 levels and decreased IL-1β, TNF-α, IL-6, and LPS concentrations in High fat diet (HFD)-induced mice, indicating a systemic anti-inflammatory response (Wang et al., 2025). In the experiments of Liu et al., the improvement of intestinal barrier function in type 2 diabetic (T2D) mice by 3,4-Dihydroxyphenylacetic acid (DHAA) and its potential mechanism were investigated. DHAA inhibited the phosphorylation of JNK and p38, and reduced the expression of MLCK proteins and the phosphorylation level of MLC, which effectively blocked the activation of the MAPK-MLCK pathway and alleviated the intestinal barrier dysfunction in T2D mice (Liu M. et al., 2022). And in Chen et al.’s study, they used both 3-(3’,4’-dihydroxyphenyl) propanoic acid (DHPA) and DHAA to investigate the effects of both on high-fat diet-induced obesity in mice and their potential mechanisms. Among the results, DHPA and DHAA significantly reduced body weight, improved glucose tolerance and insulin resistance, attenuated hepatic steatosis, and ameliorated oxidative stress in high-fat diet mice. The analysis concluded that DHPA and DHAA could affect metabolism by regulating pentose and glucuronide interactions, tyrosine metabolism, pentose phosphate pathway and tricarboxylic acid cycle (Chen W. et al., 2024).

Further microbial degradation of EC generates hydroxybenzoic acids, including protocatechuic acid (PCA), vanillic acid, meta-hydroxybenzoic acid, and GA. These metabolites have also been implicated in anti-inflammatory processes. Tan et al. reported that PCA alleviated metabolic-associated fatty liver disease (MAFLD) by inhibiting *Enterococcus faecalis* growth, reducing inflammatory cytokine production, and limiting lipid peroxidation (Tan et al., 2023). GA was shown to suppress NLRP3 inflammasome activation, decrease caspase-1 cleavage and IL-1βsecretion, and upregulate Nrf2 expression, thereby mitigating oxidative stress and inflammation ^139^. Notably, the anti-inflammatory effects of GA were abolished by Nrf2 inhibition, suggesting a Nrf2-dependent mechanism (Lin et al., 2020). Pyrogallol, another downstream metabolite, was found to enhance HO-1 expression in human umbilical cord mesenchymal stem cells (hUCMSCs), potentially strengthening their antioxidant and anti-inflammatory capabilities in an LPS-induced acute lung injury (ALI) model (Zhang et al., 2022), although direct suppression of inflammatory mediators was not explicitly demonstrated.

In addition to the anti-inflammatory effects of individual EC metabolites mentioned above, different metabolites may also exert synergistic or complementary effects by coordinating inflammatory signaling pathways through multiple targets. Examples include jointly inhibiting NF-κB activation, synergistically enhancing Nrf2-mediated antioxidant defense, and regulating the secretion of immune cytokines. In the study by Corral-Jara et al., the researchers employed a multi-omics approach and discovered that EC metabolites derived from the gut microbiota, particularly γ-pentalactone-bound metabolites, exert significant anti-inflammatory effects by synergistically regulating mRNA, miRNA, lncRNA, and protein expression. These metabolites inhibit the NF-κB inflammatory signaling pathway, reduce leukocyte adhesion and transendothelial migration, and maintain endothelial barrier integrity. This indicates that the anti-inflammatory effects of EC largely depend on the integrated action of its microbial metabolites rather than the isolated activity of the parent compound itself (Corral-Jara et al., 2021). There is now a substantial body of research on the synergistic anti-inflammatory effects of EC, but most studies focus on the synergistic anti-inflammatory actions between different polyphenolic compounds (Mechchate et al., 2021; Li et al., 2019). Research on the synergistic anti-inflammatory effects of EC metabolites remains limited, making this an area worthy of future investigation.

## 5 Limitations and future perspectives

**FIGURE 5:**
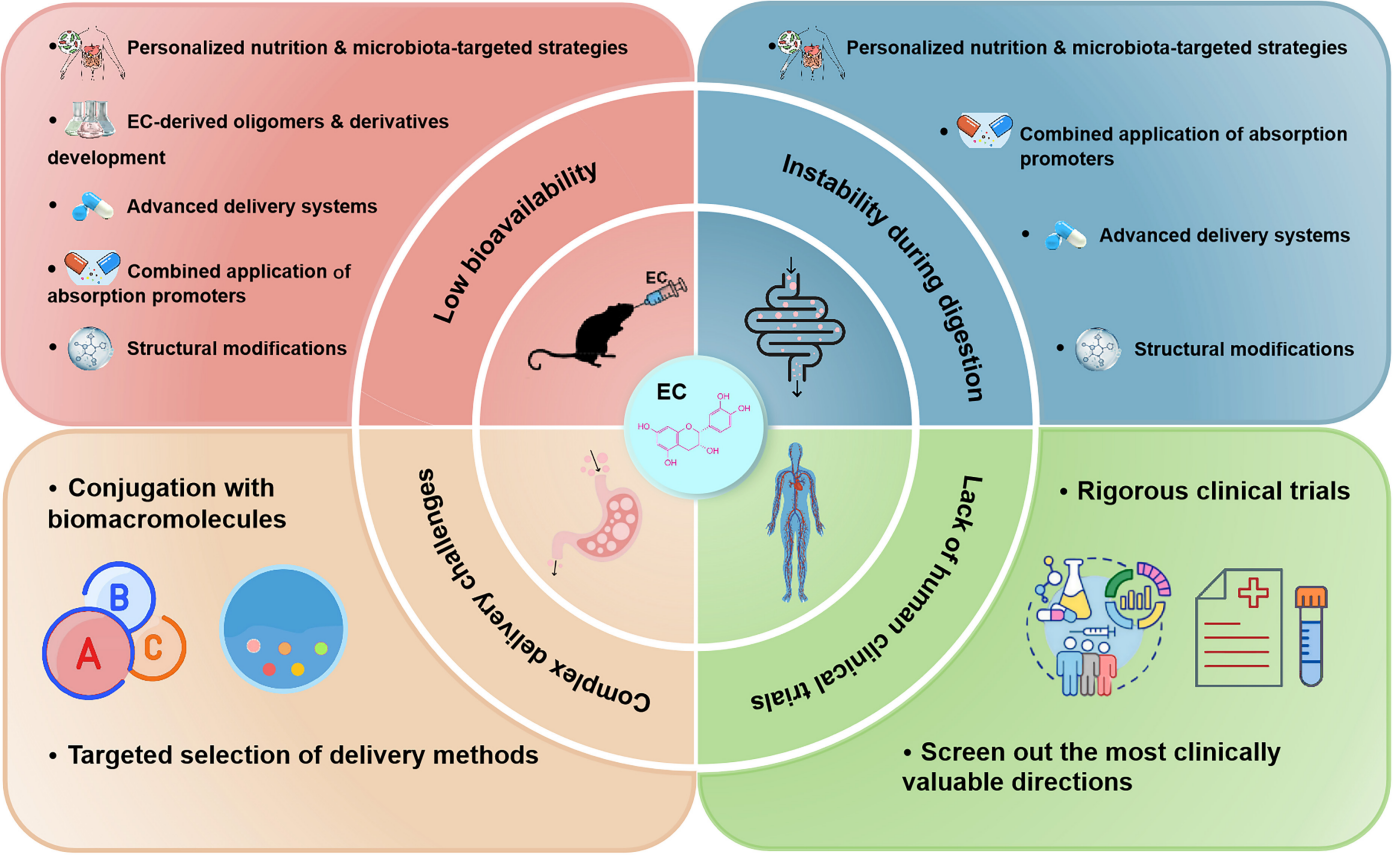
Key challenges and potential strategies for improving the clinical application of EC.

Despite its significant biological activities, EC faces major challenges in clinical application, primarily due to its low bioavailability. To address these limitations, various strategies—including structural modifications, co-administration with absorption enhancers, and advanced delivery systems—are under investigation. An overview of the major challenges and corresponding solutions is illustrated in Figure 5.

Nanotechnology-based delivery systems, including nanoencapsulation, micelles, and liposomes, offer promising solutions to improve EC’s stability, solubility, and targeted delivery (Qi et al., 2023). Encapsulation can protect EC from degradation in the gastrointestinal tract, enhance systemic absorption, and achieve controlled release (Li et al., 2025c), thereby amplifying its anti-inflammatory effects. However, current nanocarrier systems also face limitations: liposomes are prone to degradation in the acidic and enzymatic environment of the digestive tract (Zhang et al., 2023); protein- or polysaccharide-based nanoparticles involve complex preparation processes and may alter EC’s bioactivity (Kareemi and Likhitkar, 2024); and microencapsulation techniques such as spray drying often suffer from low encapsulation efficiency and imprecise release profiles (Elsayed and AbouGhaly, 2016). Future research should focus on designing more stable and efficient delivery systems, combining real-time absorption monitoring with multidisciplinary approaches to maximize EC’s therapeutic potential.

Beyond delivery innovations, structural modification of EC presents another promising strategy to enhance its bioavailability and biological efficacy. Chemical modifications such as esterification or conjugation with bioactive molecules can improve EC’s solubility, stability, and cellular uptake. For instance, introducing fatty acid chains can enhance EC’s lipophilicity, facilitating membrane transport. Pornpun et al. synthesized EC-3’-O-α-D-glucopyranoside via β-cyclodextrin-assisted glucosylation, which exhibited improved water solubility and photostability compared to native EC (Aramsangtienchai et al., 2011), although its antioxidant activity was slightly reduced. Such modifications suggest potential for EC derivatives as functional food ingredients.

In addition to individual modifications, coupling EC with other compounds has emerged as an effective strategy to enhance its stability, antioxidant, and anti-inflammatory properties. Given EC’s four phenolic hydroxyl groups, it readily interacts with lipids and proteins. Studies have shown that EC conjugation with hydrophobic proteins like soy lipophilic protein (Sun et al., 2023) or with dialdehyde starch (Yong et al., 2022) can significantly enhance its stability and antioxidant activity. However, research specifically focusing on EC conjugates for anti-inflammatory applications remains limited, with more attention historically directed toward (-)-epigallocatechin-3-O-gallate (EGCG) conjugates (Li et al., 2021, 2025a; Yong et al., 2025). Expanding the development of EC-based conjugates, particularly with biodegradable polymers, could offer new opportunities for designing inflammation-targeted therapeutics.

Furthermore, EC-derived oligomers have shown promise in enhancing anti-inflammatory effects. In a comparative study (Jeong et al., 2024), EC-derived trimers linked via methylene bridges exhibited stronger inhibitory effects on Lipopolysaccharide (LPS)-induced NO production and iNOS expression in RAW 264.7 macrophages compared to monomeric EC. Combining EC derivatives with nanodelivery systems may provide a synergistic strategy to achieve more potent, controlled, and stable anti-inflammatory responses.

Beyond bioavailability and delivery challenges, the translation of EC’s preclinical efficacy into clinical success remains a major hurdle. Many existing studies rely heavily on animal models, and the lack of proper dose extrapolation to humans complicates efficacy prediction (Shariati et al., 2019; Yang and Chan, 2018). Future clinical trials are urgently needed to determine optimal dosage regimens, establish dose-response relationships, and assess the long-term safety of EC supplementation. Only through rigorous clinical validation can EC be fully realized as a clinically viable anti-inflammatory agent.

Finally, despite recent advances in understanding the interaction mechanisms between EC and other polyphenols with gut microbiota, key functional strains and core metabolic enzymes remain incompletely characterized. Significant challenges persist, including substantial interindividual microbial diversity, incomplete elucidation of metabolic pathway mechanisms, and a lack of standardized industrial translation criteria. EC metabolism heavily relies on specific functional strains, and variations in microbial composition across individuals result in notable heterogeneity in metabolic efficiency and health benefits. Therefore, future research should integrate multi-omics technologies with functional validation models to systematically decipher EC’s key metabolic bacteria and core metabolites, construct EC metabolic pathway maps, and establish precise phenotyping and personalized intervention strategies. Concurrently, comparative studies should intensify on the bioactivity differences between EC parent compounds and their microbial metabolites. Long-term clinical validation and standardized quality control are essential to advance the “polyphenol-biotics” synergistic intervention model toward functional foods and precision nutrition applications, thereby maximizing and ensuring the sustainable utilization of polyphenol health effects (Li et al., 2025e).

## 6 Conclusion

This review provides an in-depth evaluation of EC, with a focus on its chemical basis, metabolic fate, and mechanisms of immune modulation. Beyond EC itself, increasing attention is being directed toward its gut microbial metabolites, particularly colonic-derived low-molecular-weight compounds, which may exert distinct and complementary bioactivities. However, current knowledge of their individual anti-inflammatory effects remains limited, and further mechanistic investigations are warranted to elucidate their specific roles and targets.

A particularly valuable direction for future research lies in characterizing the structure–activity relationships of these microbial metabolites and validating their efficacy in relevant disease models. Moreover, given the substantial interindividual variability in EC metabolism, integrating host and microbial factors into personalized nutrition strategies will be critical for maximizing therapeutic benefits. Addressing these challenges will not only refine our understanding of EC’s mode of action but also facilitate the development of tailored dietary interventions and precision polyphenol therapies for inflammation-related conditions.
